# A critical review of diet-related surveys in England, 1970-2018

**DOI:** 10.1186/s13690-020-00447-6

**Published:** 2020-07-20

**Authors:** Monique Campbell, Dianna Smith, Janis Baird, Christina Vogel, Emeritus Graham Moon

**Affiliations:** 1 University of Southampton, School of Geography and Environmental Science, Southampton, UK; 2 MRC Lifecourse Epidemiology Unit, University of Southampton, Southampton, UK

**Keywords:** Diet and nutrition, Researchers, Secondary data, Repeated cross-sectional and longitudinal surveys, Key features, Considerations, Diet-related surveys, Dietary assessment methods, England

## Abstract

**Background:**

Many diet-related surveys have been conducted in England over the past four to five decades. Yet, diet-related ill-health is estimated to cost the NHS £5.8 billion annually. There has been no recent assessment of the diet-related surveys currently available in England. This paper aims to fill this gap in the literature by providing researchers, especially those interested in conducting secondary (quantitative) research on diet, with a detailed overview of the major repeated cross-sectional and longitudinal surveys conducted in England over the last 48 years (1970–2018).

**Method:**

A three-stage review process was used to identify and assess surveys and synthesise the information necessary for achieving the paper’s aim. Surveys were identified using the UK Data Service, Cohort and Longitudinal Studies Enhancement Resources (CLOSER), the Medical Research Council (MRC) Cohort Directory and the Consumer Data Research Centre (CDRC) online data repositories/directories. Surveys were summarised to include a brief background, the survey design and methodology used, variables captured, the target population, level of geography covered, the type of dietary assessment method(s) used, primary data users, data accessibility, availability and costs, as well as key survey features and considerations.

**Results:**

The key considerations identified across the various surveys following the review include: the overall survey design and the different dietary assessment method(s) used in each survey; methodological changes and general inconsistencies in the type and quantity of diet-related questions posed across and within surveys over time; and differences in the level of geography and target groups captured.

**Conclusion:**

It is highly unlikely that any survey dataset will meet all the needs of researchers. Nevertheless, researchers are encouraged to make good use of the secondary data currently available, in order to conduct the research necessary for the creation of more evidence-based diet-related policies and interventions in England. The review process used in this paper is one that can be easily replicated and one which future studies can use to update and expand upon to assist researchers in identifying the survey(s) most aligned to their research questions.

## Background

Sub-optimal diet continues to be the most significant contributor to the global burden of disease, accounting for more deaths and disease than physical inactivity, alcohol consumption and smoking combined [4, 8, 10, 14]. Despite a proliferation of interventions which span decades, diet-related ill-health has been estimated to cost the National Health Service (NHS) approximately £5.8 billion annually [21]. In response to this situation, the World Health Organisation (WHO) has urged researchers to make “effective, proper and good use” of the secondary data currently available, in order to conduct the research necessary for the creation of more evidence-based diet-related policies and interventions [27]. Diet-related surveys continue to be the major source of information used by researchers and policymakers to assess dietary patterns, monitor trends over time, evaluate the success/failure of interventions and identify potential inequalities. Although the availability of diet-related survey data is limited in many European countries, England boasts several Government sponsored/endorsed repeated cross-sectional and longitudinal surveys. Surveys such as the National Diet and Nutrition Survey (NDNS), the Health Survey for England (HSE), Understanding Society, and many others, can be easily accessed online from national data repositories such as the UK Data Service, usually at little or no cost. The relative ease with which secondary data can be accessed in England at present means that now, more than ever, researchers are able to explore diet-related topics of interest and forgo what would have been an otherwise time-consuming and costly primary data collection process. Though beneficial, the analysis of secondary data still requires that researchers clearly define their research questions, critically assess diet-related surveys currently available from the outset and identify the survey(s) which best suits their unique research needs, before any data are analysed [3]. Although initially time-consuming, this type of detailed preliminary assessment is essential, as it saves time in the long run and helps to ensure the overall success of diet-related studies undertaken.

Several studies have noted general challenges and practical considerations which researchers often face when analysing diet-related data [1, 12, 13, 16, 26]. Examples of these include: the unavailability of consistent, nationally representative diet-related data, different dietary assessment methods used in surveys and the tendency for surveys to capture data on single food groups/nutrients (such as fruits and vegetables) as opposed to a variety of foods. Rippin et al. [20] previously assessed the current status of nationally representative surveys in Europe. However, the authors of that study only focused on the 53 countries in the WHO European region and not England specifically. Overall, very few studies have outlined and discussed diet-related surveys conducted in England, their characteristics, possible benefits and some of the practical and unique considerations researchers should note when trying to decide the survey dataset(s) most aligned to their research question(s).

This paper is not a systematic review but, rather, a secondary data review which aims to fill a gap in the literature by providing researchers, especially those interested in conducting secondary (quantitative) research on diet and with limited time and resources, with a detailed overview and summary of the strengths and weaknesses of the major repeated cross-sectional and longitudinal surveys conducted in England over the last 48 years (1970–2018). Surveys identified and discussed in this review should not be interpreted as being capable of meeting all the needs of researchers involved/interested in diet-related research. Instead, this review will provide a brief background on some of the major diet-related repeated cross-sectional and longitudinal surveys conducted in England over the past four decades, the survey design and methodology used, variables captured, the target population, level of geography covered, the type of dietary assessment method(s) used, primary users of the data and information related to data accessibility, availability and costs. Additionally, key survey features which could benefit some researchers in answering their particular research question(s) will be highlighted, as well as some practical considerations which should be acknowledged before selecting and analysing data. To the best our knowledge, this is the first paper to provide this type of detailed information on a current snapshot of major repeated cross-sectional and longitudinal diet-related surveys in England. This information could serve as a template or a quick guide which researchers can refer to as a starting point to identify existing diet-related surveys, assess potential survey benefits/issues and the possible impact (positive or negative) this could have on their research. This information will enable researchers to develop separate work-around strategies (where necessary) to suit their unique research needs and will save them time and resources than if it were necessary to compile this information from scratch.

## Methods

**Fig. 1:**
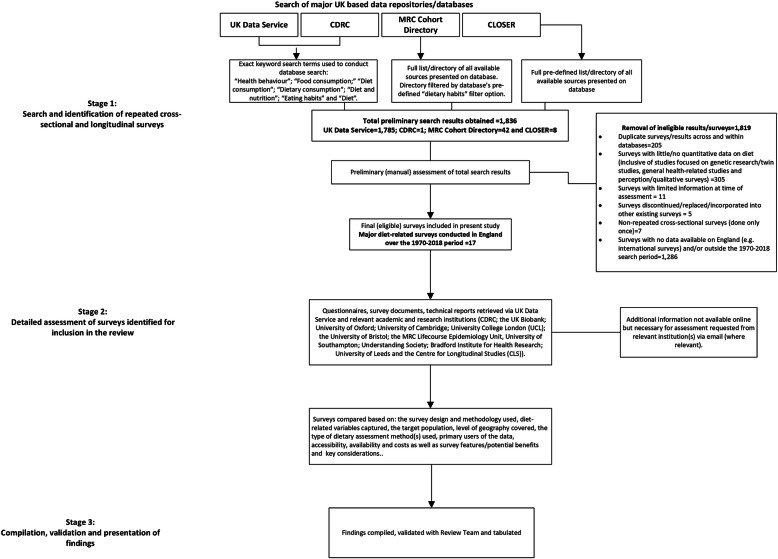
Three-stage research and review process used to identify and assess surveys and synthesise findings

Preliminary meetings were held with all members of the paper’s Review Team (MC, DS, JB, GM and CV) to discuss the scope, eligibility criteria and analytic strategy of this review. The decision was to include repeated cross-sectional and longitudinal surveys, where quantitative information on diets in England was collected over the 1970–2018 period. A three-stage review process was used to identify, assess and synthesise the information necessary for achieving this paper’s aim (Fig. 1).

Stage one of the review process (Fig. 1), involved the identification of all major repeated cross-sectional and longitudinal health, diet-related surveys, conducted in England over the period from January 1970 to December 2018. This assessment period (48 years) was thought to be an adequate time span in which a sufficient number of longstanding and current survey datasets (especially longitudinal surveys) could be captured. Surveys were identified using the four major online directories currently available and used by researchers in the UK, namely: the UK Data Service, the Medical Research Council (MRC) Cohort Directory, Cohort and Longitudinal Studies Enhancement Resources (CLOSER) and the Consumer Data Research Centre’s (CDRC) online directory. These four online directories were selected because they provided a comprehensive list of all surveys conducted within the UK over time, a summary of the survey design, variables captured within datasets, links to survey documentation and where relevant, the institutions (academic and research) ultimately responsible for managing and disseminating data.

The search strategy used to identify initial survey results varied, based on how each of the four databases were inherently structured. For the UK Data Service and the CDRC databases, an exact keyword search for “Health behaviour”; “Food consumption;” “Diet consumption”; “Dietary consumption”; “Diet and nutrition”; “Eating habits” and “Diet” was conducted. This was done to ensure that a wide variety of surveys, especially those not directly associated with diet, but which captured aspects of diet-related behaviours, would have been initially identified. The MRC Cohort Directory presented a full list of all major cohort (longitudinal) studies conducted in the UK, from which diet-related surveys relevant to this review were identified using the database’s pre-defined “Dietary Habits” topic filter option. CLOSER was strictly focused on eight longitudinal surveys (the Hertfordshire Cohort Study, 1946 and 1970 British Cohort Study, 1958 Child Development Study, Avon Longitudinal Study of Parents and Children, Southampton Women’s Survey, Understanding Society and Millennium Cohort Study) which captured persons born throughout the 20th and 21st centuries. All eight affiliated surveys were listed in the “Our Studies” section of the CLOSER database, which meant there was no need to filter or conduct any keyword searches. In total, 1836 preliminary results were obtained across the four databases, of which 97% (1785 results) were from the UK Data Service.

Preliminary search results obtained were manually assessed by the Review Team (MC, DS, JB, GM and CV) to filter out duplicates (205 of the 1836 total preliminary results) and surveys which did not meet the paper’s eligibility criteria (1614 out of the 1836 total preliminary results). Ineligible surveys included: discontinued surveys, non-repeated cross-sectional surveys conducted only once, surveys which although diet-related, had no data for England (e.g. international studies or studies focused on a particular UK constituent country such as Scotland only), surveys which fell outside the 1970–2018 search period, surveys which had little or no quantitative diet-related data (e.g. qualitative/perception studies, gene/twin studies, general health studies with no diet-related data) and surveys which could not have been properly assessed due to limited documentation at the time of assessment. The removal of duplicate and ineligible surveys (1819 results omitted), reduced the results from 1836 to 17 surveys eligible for inclusion in the current review (Fig. 1).

In Stage two of the review process, questionnaires, documents and technical reports for the 17 eligible surveys were retrieved online from the UK Data Service and the official website of the responsible academic and research institutions. Academic and research institutions included: the CDRC; UK Biobank; University of Oxford; University of Cambridge; University College London (UCL); University of Bristol; the MRC Lifecourse Epidemiology Unit, University of Southampton; Understanding Society; the Bradford Institute for Health Research; University of Leeds and the Centre for Longitudinal Studies (CLS). Where necessary, follow-up emails were sent directly to the UK Data Service and institutions to collect additional information not available on official websites. Documents (inclusive of questionnaires used across survey waves/periods) received either from websites or via email were thoroughly reviewed in order to identify: the survey design and methodology used, diet-related questions/variables captured, the target population, level of geography covered, the type of dietary assessment method(s) used, primary users of the data, accessibility, availability and data costs, as well as the key survey features/potential benefits and key considerations for each survey.

Finally, Stage three involved the compilation of findings, which were cross-validated with all members of the Review Team (MC, DS, JB, GM and CV) and tabulated (see Table 1) in order to capture the detailed information on all 17 surveys in an easy to understand and user-friendly manner.

**Table 1:** Summary/review of 17 major repeated cross-sectional and longitudinal surveys conducted in England over the January, 1970 to December, 2018 period

Repeated cross-sectional surveys		
	**Living Cost and Food Survey (LCFS)**	**Actives Lives Survey (ALS)**
**Survey Background**	The LCFS (formerly known as the Expenditure and Food Survey (EFS) prior to 2008) is the UK’s premier household expenditure survey, which captures information on the spending patterns and cost of living across the UK.	The ALS, which replaced the Active People Survey in November, 2015, is a sport and recreation survey which measures physical activity levels of over 198,000 persons living across England.
**Survey Design and Methodology**	Annual repeated cross-sectional survey. Sample selected using multi-stage stratified random sampling with clustering. Household addresses with small user postcodes are randomly selected from the Royal Mail’s postcode address file (PAF). Face-to face interviews (individual and household questionnaires administered) and 2-week self-reported expenditure diaries completed by all members of the household, aged 16 years and over. Simplified expenditure diaries are completed by children 7 to 15 years old.	Annual repeated cross-sectional survey. Multi-stage stratified random sample. Each year, approximately 198,250 persons are targeted for inclusion in the survey. Household addresses are randomly selected from the Royal Mail’s postcode address file (PAF) and letters sent inviting up to two adults (16 years and over) per household to complete a questionnaire online or via post (for persons without internet access). Participants are rewarded with a £5 voucher from a range of retailers. During the adult survey, persons are asked if there are any 14–15-year olds in their household. Children aged 14–15 who ae interested and receive parental consent to participate in the study are contacted and asked to complete a young person questionnaire.
**Target population and level of geography covered**	Families/households within the UK (England, Scotland, Northern Ireland and Wales). Data for England are available at the national and Government Office Region (GOR) level. Local authority level data can be made available upon request and approval by the UK Data Service.	Individuals 14 years and older living in England during the 2015–2016 and 2016–2017. Data are available for the Government Office Region (GOR), County Sport Partnerships, Counties and Local Authority District level. The survey was designed to achieve a minimum annual sample size of 500 for each local authority, with the exception of the City of London and Isles of Scilly, in which the target sample size was 250.
**Type of dietary assessment used**	Household food expenditure data captured in the Family Food Module of the survey are used as a proxy measure for food consumption.	Single 24-h screener/brief/shortened instrument (fruit and vegetable only) completed online or via post.
**Primary users of diet-related data**	Academics/Researchers and several Governmental Departments. The Family Food Module of the LCFS is primarily used by the Department for Environment Food and Rural Affairs (Defra) to monitor food consumption and to produce the annual Family Food Report (a report which provides estimates of nutrient content and statistics on household food purchases by food type).	Academics/Researchers, Local Authorities, Public Health England (PHE)
**Data Accessibility/Availability**	Data accessible through the UK Data Service. Data currently available for the 2008–2017/18 period	Data accessible through the UK Data Service. Data currently available for the 2015–2016 and 2016–2017 survey periods.
**Types of variables captured**	Socio-demographic information (age, sex, occupation, education), GOR, local authority level geography, data garnered from 2-week expenditure diary (expenditure on energy, bills, utilities and food).	Socio-demographic information (age, sex, employment) and health measures such as obesity and fruit and vegetable consumption over a 24-h period.
**Cost to access**	Not applicable	Not applicable
**Key features/potential benefits**	1. Nationally representative annual survey with relatively large sample size (approximately 5000 households each year) 2. Two (2) week expenditure diaries (completed by each member of the household 16 years and over) detailing purchased quantities of food and drink are used to estimate food consumption in England. 3. Possible to make comparisons between low and high-income households.	1. Large sample size and a nationally representative sample of the English population. 2. Although focused on sport and recreation, the survey also includes data on fruit and vegetable consumption. 3. The availability of local authority data makes it possible to analyse dietary consumption below the regional (GOR) level.
**Key considerations**	1. Difficult to compare data prior to 2008 as a different survey methodology was used for the previous EFS. 2. Survey designed to capture household expenditure on food and quantities of food and drink purchased. The survey does not capture foods actually consumed by individuals.	1. The survey only captures self-reported fruit and vegetable consumption over a single 24-h period. 2. Difficult to compare data prior to 2015 as a different survey methodology was used for the previous Active People Survey.
	**National Diet and Nutrition Survey (NDNS)**	**Health Survey for England (HSE)**
**Survey Background**	The NDNS was originally established in 1992 as a series of four separate cross-sectional surveys, capturing information on: children ages 1 ½ -4 ½ years (1992–1993), young people 4–18 years old (1997), adults 19–64 years old (2000–2001) and persons 65 years and over (1994–1995). In 2008, the new NDNS Rolling Programme (RP) was introduced as a nationally representative repeated cross-sectional survey which captures information on the type and quantity of foods and beverages consumed by 1000 persons (500 adults and 500 children) annually in the UK.	The HSE is an annual survey used to monitor and assess changes in the overall health and lifestyle of persons living within England.
**Survey Design and Methodology**	Annual repeated cross-sectional survey. Multi-stage stratified random sample. Face-to face interviews conducted with respondents to capture food preparation, smoking and drinking habits. Self-completed 4-day food diaries are completed by persons 12 years and older and parents and/or carers are asked to complete food diaries for children 11 years and younger. Anthropometric measurements and blood and urine samples collected via nurse interview.	Annual repeated cross-sectional survey. Multi-stage stratified random sample. Face-to face interviews, self-completed questionnaires and a follow-up nurse visit carried out to collect anthropometric measurements and blood samples.
**Target population and level of geography covered**	Individuals 1 ½ years and older, residing in private households in the UK. Data for England are available at the national and Government Office Region (GOR) level.	Adults (defined as persons 16 years and older) and children (0–15 years old) living in private households in England. Data available at the national, Government Office Region (GOR) and Strategic Health Authorities level. Local authority level data only available upon request and approval by NatCen Social Research at a cost.
**Type of dietary assessment used**	Four (4) day food diary	Food frequency questionnaire (FFQ) used prior to 2009. Single 24-h screener/brief/shortened instrument (fruit and vegetable only) used since 2009.
**Primary users of diet-related data**	Academics/Researchers, policymakers, UK Health Departments, Scientific Advisory Committee on Nutrition’s (SACN), Food Standards Agency (FSA) and several Governmental Departments.	Academics/Researchers, policymakers, the Department of Health & Social Care, Public Health England (PHE), NHS England, other NHS bodies, Local Authorities, charities and voluntary organisations. Data used to track the national achievement of the 5-A-Day, fruit and vegetable target.
**Data Accessibility/Availability**	Data for the NDNS RP are accessible through the UK Data Service. Data currently available for the 2008–2016/17 period (survey wave 1–9).	Data are accessible through the UK Data Service. Data currently available for the 1991–2017 period.
**Types of variables captured**	Socio-demographic information (age, sex, occupation, education), GOR and all foods and beverages consumed over a 4-day period.	Socio-demographic information (age, sex, occupation, education), GOR, general health, height and weight measurements and fruit and vegetable consumption.
**Cost to access**	Not applicable	No cost to access GOR level data but lower level geography (e.g. local authority level) can be accessed at a minimum cost of £1000.
**Key features/potential benefits**	1. Availability of annual food consumption data at the national level and 2. Detailed information available on all foods and beverages actually consumed by individuals over a 4-day period using the food diary method.	1. Nationally representative annual survey with large sample size of approximately 10,000 individuals (8000 adults and 2000 children). 2. Data captured could be used to explore relationships between diet (specifically fruit and vegetable consumption), obesity and associated chronic diseases.
**Key considerations**	1. Relatively small annual sample size compared to larger cohort studies which employ methods which are less tedious than the food diary method. 2. Difficult to compare data prior to 2008 with NDNS RP data, as a different survey methodology was used previously. This makes it difficult for comparisons to be made across the survey waves and for changes in diet to be assessed over time.	1. Significant changes (e.g. the complete omission of the fruit and vegetable module in the 2012 survey wave) have been made to the type of diet questions asked, which makes it difficult for comparisons to be made across the survey waves and for changes in diet to be assessed over time.
**Food and You Survey**		
**Survey Background**	Food and You is a random probability survey commissioned by the Food Standards Agency (FSA) every 2 y. The survey captures information on public attitudes and self-reported knowledge as it relates to food safety, production and other food-related issues.	
**Survey Design and Methodology**	Bi-annual repeated cross-sectional survey. Multi-stage stratified random sample. Face-to face interviews conducted with adults, defined as persons aged 16 years and over.	
**Target population and level of geography covered**	Adults (16 and over) residing in private households the UK. Data for England are available at the national and Government Office Region (GOR) level.	
**Type of dietary assessment used**	Food frequency questionnaire (FFQ) conducted at each wave of the survey	
**Primary users of diet-related data**	Academics/Researchers, policymakers and several Governmental Departments, particularly the Food Standards Agency (FSA)	
**Data Accessibility/Availability**	Data are accessible through the UK Data Service. Data currently available for the five survey waves completed to date: 2010, 2012, 2014, 2016 and 2018.	
**Types of variables captured**	Socio-demographic information (age, sex, occupation, education, household income), GOR, frequency of consumption of foods such as beef, poultry, burgers, ready meals, diary, fruits and vegetables.	
**Cost to access**	Not applicable	
**Key features/potential benefits**	1. Nationally representative survey with sample size of about 3000–3500 individuals every 2 years 2. Besides data collected via FFQs, the survey also captures respondents’ knowledge of current dietary recommendations and perceptions of what constitutes a healthy and balanced diet.	
**Key considerations**	1. Changes made to diet-related questions asked over the years, makes it difficult for comparisons to be made across the survey waves and for changes in diet to be assessed over time.	
**Longitudinal surveys**		
	**Southampton Women’s Survey (SWS)**	**Born in Bradford (BIB)**
**Survey Background**	The SWS was established between 1998 and 2002 with the primary aim of measuring non-pregnant women aged 20–34 years living in Southampton (England) and to follow-up members of the cohort who subsequently became pregnant. The study’s major aim was to examine the effect of diet and lifestyle factors on the health of mothers and their children throughout the lifecourse.	BIB is a study which tracks the health of over 13,500 children (and their parents) born at the Bradford Royal Infirmary between March 2007 and December 2008. The study tracks the health of these children from pregnancy throughout childhood and into adulthood.
**Survey Design and Methodology**	Longitudinal Birth Cohort study. Pre-pregnancy home visits were made to 12,583 non-pregnant women (who were 20–34 years old during the 1998–2002 period) who resided in Southampton, England and surrounding areas. Pre-pregnancy food diaries were completed by participants and face-to-face interviews and blood samples were taken by a research nurse. Follow-up nurse visits were made to 3158 women who became pregnant and delivered a live born child; blood samples taken, and follow-up interviews conducted. Participants were asked to keep a food diary during early and late pregnancy. Follow-up surveys were conducted when children were 6 and 12 months and 3, 6–7, 8–9 and 11–13 years old.	Longitudinal Birth Cohort study. Women who planned to be give birth during the 2007–2011 period were recruited and baseline data on socio-economic status, ethnicity and family trees, diet, physical and mental health were collected from 12,453 women at 26–28 weeks of pregnancy. Baseline data were also collected from 3448 partners of recruited mothers. Follow-up self-administered questionnaires were completed by partners at 6 and 12 months. Follow-up home visits were made with 2 sub-groups within the cohort when children were 6, 12, 18 months and 2, 3 and 4 years old to collect information on growth trajectories, risk factors for childhood obesity and exposures to asthma and atopy. Follow-up waves are heavily dependent on the level of funding available.
**Target population and level of geography covered**	12,583 non-pregnant women aged 20–34 years during the 1998–2002 period, living in Southampton (South East of England) and surrounding areas; 3158 women who became pregnant and delivered a live born child subsequent to recruitment and their children.	Pregnant women (26–28 weeks) who delivered babies at the Bradford (North England) Royal Infirmary, fathers of the children and the children born to recruited mothers. Geographical area captured: Bradford (North of England)
**Type of dietary assessment used**	Interviewer administered FFQ and 24-h recall conducted at each survey wave, food diaries completed by mothers at pre-pregnancy, early pregnancy and when children were 3 years old and 24-h diet recalls administered when children were 6 months old.	Food frequency questionnaire (FFQ) at each wave of the survey.
**Primary users of diet-related data**	Academics/Researchers, Local Authority.	Academics/Researchers, Local Authority, National Health Service (NHS).
**Data Accessibility/Availability**	Data accessible through the MRC Lifecourse Epidemiology Unit, University of Southampton. Data available for women before pregnancy (1998–2002) and during early and late pregnancy. Data for children are available for 6 and 12 months, 3, 6–7, 8–9 and 11–13 years old.	Data (and details regarding survey data currently available) accessible through the Bradford Institute for Health Research.
**Types of variables captured**	Socio-demographic information (age, sex, occupation, employment, education), general diet, dietary changes and a 100-point FFQ asking the frequency of consumption in the last 3 months of fruits, vegetables, potatoes, rice, soft drinks, dairy, bread and a host of other foods across the various food groups.	Socio-demographic information (age, sex, occupation, employment, education) and a more than 100-point FFQ asking the frequency of consumption in the last 2–3 months of fruits, vegetables and a host of other foods across the various food groups.
**Cost to access**	Not applicable	Not applicable
**Key features/potential benefits**	1. Food consumption data available for a wide variety of foods. 2. Cohort study data can be used to track changes over time. 3. Availability of pre- and post-pregnancy data.	1. Food consumption data available for a wide variety of foods 2. Cohort study data can be used to track changes over time. 3. Bradford has a large ethnic community and so the study captures ethnic minority groups which are usually underrepresented
**Key considerations**	1. Study not representative of English population; only focuses on Southampton (South of England). 2.The study only focuses on women and their children over time. 3. Complete data on children are not available for the entire cohort at each age of follow-up.	1.Study not representative of English population; only focuses on Bradford (North of England) 2. Changes made to diet-related questions across the survey waves may make it difficult to make comparisons over time. 3. Follow-up waves are heavily dependent on the level of funding available.
	**Understanding Society**	**British Cohort Study 1970 (BCS70)**
**Survey Background**	Understanding Society is an annual large-scale, multi-topic longitudinal cohort study established to understand social and economic changes in the UK at the individual and household level.	The BCS70 is a large national longitudinal birth cohort study which tracks over 17,000 persons born in England, Scotland and Wales in a single week in 1970. The study has gathered information related to the health, social, economic and educational development of participants.
**Survey Design and Methodology**	Annual Longitudinal/panel/cohort study. Multi-stage stratified random sample. The first wave was conducted in 2009 when over 40,000 households were selected. Since then, follow-up interviews have been conducted with the same individuals every 12 months. At each survey wave, one member of the household is asked to complete a household questionnaire and each person 16 years and older is interviewed and asked to complete a separate (self-completed) questionnaire. Members of the household aged 10–15 years (young people) are also asked to complete a separate (self-completed) paper or web-based/online questionnaire. Web-based surveys were introduced in wave 7 (2016) of the survey.	Longitudinal Birth Cohort study. All children born in England, Scotland and Wales in 1970 were recruited and eight follow-up surveys have been conducted to date. Follow-up interviews were done when children were 5, 10, 16, 26, 30, 34, and 42 years of age (in 2012). Although data are not currently available, a follow-up survey was conducted at age 46 (in 2016) and information is currently being processed. In the 2004 study (age 34) cohort members were given a basic skills (numeracy and literacy) assessment test and a self-completion questionnaire to complete.
**Target population and level of geography covered**	Individuals living within over 40,000 households in the UK. Data for England are available at the national and Government Office Region (GOR) level. Local authority level, Westminster Parliamentary Constituencies, Local Education Authorities and Travel to Work Areas are available upon request and approval by the UK Data Service under its Special License Agreement.	Children born in England, Scotland and Wales in a single week in 1970.
**Type of dietary assessment used**	Short food frequency screener/brief instrument which primarily captured fruit and vegetable consumption.	4-day food diary and a 24-h diet recall included in 1986 wave of survey. Online diet diary also included in the 2016 wave, when respondents were 46 years old.
**Primary users of diet-related data**	Academics/Researchers.	Academics/Researchers.
**Data Accessibility/Availability**	Data accessible through the UK Data Service. Data currently available for the 2009–2018 period (survey wave 1–9)	Data accessible through the UK Data Service. Data currently available for the 1975–2016-18 survey period.
**Types of variables captured**	Socio-demographic information (age, sex, education, family, social life), self-reported health, type of milk, bread usually consumed, daily and weekly consumption of fruits and vegetables.	Socio-demographic information (age, sex, occupation, education). Consumption of fruits, vegetables, meat, dairy, soup, potatoes, biscuits, crisps, fizzy drinks, sweets and ice-cream consumed over a 24-h period. All foods consumed over a 4-day period in 1986 (paper-based food diary) and in 2016 (online food diary) when respondents were 46 years old.
**Cost to access**	Not applicable	Not applicable
**Key features/potential benefits**	1. Large sample size, nationally representative and conducted annually. 2. Cohort study data can be used to track changes over time.	1. Large sample size and nationally representative 2. Cohort study data can be used to track changes over time. 3. Detailed information on all foods consumed by participants over several days were captured in food diaries conducted in the 1986 and 2016 wave of the survey.
**Key considerations**	1. Very few diet-related questions included in the study (fruit and vegetable consumption, dairy, bread). Questions posed in the main questionnaire primarily focused on the type of bread and milk consumed and portions of fruits and vegetables consumed in a typical week. 2. Differences in the number and types of diet-related questions asked across survey waves could make it difficult for comparisons to be made over time.	1. Food diary data for the 1986 and 2016 wave are being cleaned and the expected date of release is undetermined 2. Changes made to diet-related questions across survey waves could make it difficult for comparisons to be made over time.
	**Avon Longitudinal Study of Parents and Children (ALSPAC)**	**UK Women’s Cohort Study (UKWCS)**
**Survey Background**	ALSPAC also known as the Children of the 90s Study, is a study which tracks the health and well-being of 14,400 families living within the Bristol area.	The UKWCS is a large-scale cohort study which explores the relationship between diet (including foods, nutrients and supplements) and health outcomes such as cancer, cardiovascular disease and obesity amongst over 35,000 middle aged women in the UK.
**Survey Design and Methodology**	Longitudinal Birth Cohort study. Study posters were disseminated, and local community midwives discussed the study with women with expected deliveries between April 1991 and December 1992. Persons who contacted the study team were included in the study. Baseline data were captured during pregnancy and follow-up assessments carried out when children were 4 weeks to 24 years of age. Self-completed postal questionnaires were completed by mothers, children and teachers (of children) and clinical assessment visits were carried out at different stages of the study.	Longitudinal Cohort study. Direct mail questionnaires were sent by the World Cancer Research Fund to persons, particularly women, living in England, Scotland and Wales, listed on direct mailing lists. Female survey responders aged 35–69, who self-identified as vegetarian or non-red meat eaters were included in the study. Baseline data were collected during the 1995–1998 period and follow up (known as phase 2 of the study) was done during the 1999–2002 period. Several sub-studies have been carried out over the years. For instance, an iron status sub-study in 2000–2002, a snacking study in 2006 and a pilot study to test a web-based 24-h dietary assessment tool in 2014.
**Target population and level of geography covered**	All women pregnant during 1990–1992, who resided in Bristol/Avon Health Authority and surrounding areas, their partners and all children born out of these pregnancies. Geographical area captured: Bristol and surrounding areas (South West of England)	Middle aged women (aged 35–69 at recruitment) living in England, Scotland and Wales, who self-reported as being vegetarian or non-red meat eaters. Geographical area captured: England, Scotland and Wales and English regions. Regions included in the study’s data set can be easily converted to Government Office Region (GOR) categories
**Type of dietary assessment used**	Food frequency questionnaires (FFQs). Food diaries were completed by parents when children were 7, 10 and 13 years of age.	Food frequency questionnaire (FFQ); a 4-day food diary (completed during the follow up study in 1999–2002) and a 24-h web-based diet recall assessment pilot in 2014.
**Primary users of diet-related data**	Academics/Researchers.	Academics/Researchers.
**Data Accessibility/Availability**	Data (and details regarding survey data currently available) accessible through the University of Bristol	Data (and details regarding survey data currently available) accessible through the Consumer Data Research Centre
**Types of variables captured**	Socio-demographic information (age, sex, occupation, employment, education), consumption of fruits, vegetables and a host of other foods which vary across the survey waves.	Socio-demographic information (age, sex, occupation, education), food consumption data captured from FFQs and food diaries conducted at different survey waves.
**Cost to access**	Minimum cost of £2715 to access	Not applicable
**Key features/potential benefits**	1. Large sample size. 2. Cohort study data can be used to track changes over time.	1. Large sample size. 2. Cohort study data could be used to track changes over time. 3. Availability of food diary data provides detailed information on all foods consumed by participants.
**Key considerations**	1. Costly to access. 2. Study not representative of English population; only focuses on Bristol and surrounding areas (South West of England). 3. Changes made to diet-related questions across survey waves could make it difficult for comparisons to be made over time	1. Food diaries completed in phase 2 of the study (1999–2002) and diaries completed during the 2014 online pilot study were still being processed at the time of this assessment. As such, these data are not available, and the date of release is undetermined 2. Study not representative of the English population. Participants were mostly vegetarian, middle aged, middle class, white women who volunteered to be a part of the study during the late 1990s 3. Changes made to diet-related questions across the survey waves may make it difficult to make comparisons over time.
	**Whitehall II Study**	**Millennium Cohort Study (MCS)**
**Survey Background**	The Whitehall II study is a cohort study conducted to assess the causes of social inequalities in health in England.	The MCS is a large national longitudinal birth cohort study which tracks 19,000 children born in the UK during 2000–2001, from childhood into adulthood.
**Survey Design and Methodology**	Longitudinal Cohort study. A cohort of 10,308 middle-aged persons (3413 females and 6895 males, aged 35–55 years old) who worked in the London offices of 20 Whitehall departments in 1985–1988 were included in the study. During the 2015–2016 period, research clinics were established in London, Bristol, Birmingham and Liverpool to allow persons (especially retired persons) now living within these and surrounding areas to be a part of the study and reduce the level of attrition. Members of the cohort were invited to attend a clinic research screening every 5 years and a postal survey sent to participants between clinic phases. Overall, data has been collected over 12 waves, from 1985 to 1988 to 2015–2016	Longitudinal Birth Cohort study. Multi-stage stratified random sample. The sample consisted of all children born (live births) over 12 months (from 1 September 2000 in England and Wales and for 59 weeks from 22 November 2000 in Scotland and Northern Ireland). Six surveys have been conducted to date, capturing information when children were 9 months and 3, 5, 7, 11 and 14 years of age (in 2015). Although data are currently unavailable, the 7th wave was conducted in 2018 captures children at age 18. A combination of data collection methods has been used. These include face-to-face interviews, self-completed questionnaires; psychological measurements, observation; time use diaries and physical measurements.
**Target population and level of geography covered**	Middle-aged persons who worked in the London offices of 20 Whitehall departments in 1985–1988.	Children born in the UK (England, Scotland, Northern Ireland and Wales) during 2000–2001. Data for England are available at the national and Government Office Region (GOR).
**Type of dietary assessment used**	Food frequency questionnaire (FFQ)	Food frequency questionnaire (FFQ)
**Primary users of diet-related data**	Academics/Researchers.	Academics/Researchers.
**Data Accessibility/Availability**	Data accessible through the University College London. Data available for waves 1–12 (1986–2016)	Data accessible through the UK Data Service. Data currently available for the 2001–2015 survey period.
**Types of variables captured**	Socio-demographic information (age, sex, occupation, employment, retirement, education, income), self-reported health and frequency of consumption in the last 12 months of fruits, vegetables, meat, fish, soups, sauces, spreads, eggs, dairy products, fats, bread, pasta, potato, rice, sweets and snacks were consumed.	Socio-demographic information (age, sex, occupation, employment, education of parents), consumption of fruits and vegetables and other foods such as bread, milk, sugary drinks and fast foods.
**Cost to access**	Not applicable	Not applicable
**Key features/potential benefits**	1. Food consumption data available for a wide variety of foods. 2. Fairly large sample size across the 12 waves (10,308 in 1985–1988 to 5632 in 2015–2016). 3. Cohort study data can be used to track changes over time.	1. Large sample size and nationally representative. 2. Cohort study data can be used to track changes over time. 3. Children were asked to state their consumption of fruits and vegetables and other foods such as bread, sugary drinks and fast food at age 14.
**Key considerations**	1. Study not representative of English population. Study focused on middle-aged civil servants. 2. Changes made to diet-related questions across the survey waves may make it difficult to make comparisons over time. 3. Based on the current age-group of participants, the study is now primarily focused on issues surrounding population ageing.	1. Cohort members are still very young, which currently limits the assessment of diet by age/over lifecourse. 2. Changes made to diet-related questions across survey waves could make it difficult for comparisons to be made over time.
	**European Prospective Investigation into Cancer and Nutrition (EPIC Norfolk/Oxford)**	**UK Biobank**
**Survey Background**	EPIC is a large cohort study which aims to examine diet as a risk factor for cancer and other chronic diseases amongst over 80,000 middle aged persons in the UK.	The UK Biobank is a large-scale longitudinal study which follows 500,000 middle-aged persons across the UK to investigate the association between diet and a range of diseases such as cancer, heart disease, stroke, diabetes and dementia.
**Survey Design and Methodology**	Longitudinal Cohort study. EPIC Oxford: 65,000 persons from the general population were recruited between 1993 and 1999 via EPIC nurses in GP practices in Greater Manchester, Oxfordshire and Buckinghamshire, England. Postal questionnaires were also sent to members of the Vegetarian Society of the UK and Vegan Society, and study information distributed through health magazines and shops, to capture persons located across the entire UK. Follow-up surveys were conducted 5, 10 and 15 years later. EPIC Norfolk: Invitations were sent to all 40–79-year olds on collaborating GP listings. Over 30,000 persons within Norwich and surrounding areas (East of England) were recruited over the 1993–1997 period. Participants were followed up at 18 months, 3, 13 and 20 years after recruitment. A combination of data collection methods was used for both studies (nurse interview to collect anthropometric measurements and blood samples, self-completed questionnaires (on physical activity) and record linkages via hospital diagnoses, death certification and cancer registration	Longitudinal Cohort study. Population-based registers such as those held by the National Health Service (NHS) were used as a sampling frame to identify persons living within proximity to study assessment centres. Each assessment centre aimed to recruit as many persons within the target population. Baseline data (for the 2006–2010 period) were collected at assessment centres, where self-reported baseline questionnaires were used to collect health and lifestyle-related data and interviews conducted to collect physical measurements and biological samples. A follow up survey was conducted in 2011–2012.
**Target population and level of geography covered**	EPIC Oxford: Men and women 35 years and over (at recruitment) who lived in Greater Manchester, Oxfordshire and Buckinghamshire in England and vegetarians /vegans located across the UK. EPIC Norfolk: Men and women aged 40–79 (at recruitment) who lived in Norwich and surrounding towns and rural areas.	Middle-aged males and females (persons aged 40–69 during the 2006–2010 period) who lived within a 10-mile radius of 35 study centres strategically located across England, Wales and Scotland.
**Type of dietary assessment used**	Food frequency questionnaire (FFQ) and a 7-day food diary (completed at recruitment and at the 2nd wave of the study)	Food frequency questionnaire (FFQ) with foods related to increased cancer risk conducted at baseline. Web-based 24-h recall repeated on four occasions over a 16-month period.
**Primary users of diet-related data**	Academics/Researchers.	Academics/Researchers.
**Data Accessibility/Availability**	The EPIC Oxford study is accessible through the University of Oxford and EPIC Norfolk through the University of Cambridge. Details on current data availability accessible from both institutions.	Data accessible through the UK Biobank. Data available (at the time of assessment) for the 2006–2010 (baseline) and 2011–2012 period.
**Types of variables captured**	Socio-demographic information (age, sex, occupation, education), food consumption data captured from FFQs and food diaries conducted at different survey waves.	Socio-demographic information (age, sex, employment) and fruits, vegetables, meat, dairy and a host of other foods consumed (total of over 200 foods) over a 24-h period.
**Cost to access**	Not applicable	Minimum £2000 to cover application and data access cost. Possibly reduced cost of £500 for research students (subject to review and approval).
**Key features/potential benefits**	1. Large sample size. 2. Cohort study data can be used to track changes over time. 3. Availability of food diary data (at recruitment and wave 2) which provides detailed information on foods consumed by participants.	1. Large sample size. 2. Cohort study data can be used to track changes over time. 3. Detailed information on foods consumed by participants over repeated days (repeated 24-h diet recalls).
**Key considerations**	1. Study not representative of English population. Focused on middle-aged persons living in Norwich, Greater Manchester, Oxfordshire and Buckinghamshire who were in some instances selected via purposive sampling. 2. 50% of participants were vegetarians/vegans. 3. Changes made to diet-related questions across the survey waves may make it difficult to make comparisons over time	1. Study not representative of English population. Focused on middle-aged persons from less-deprived areas (based on the target population). 2. The baseline survey captured some aspects of diet consumption but was not as comprehensive as the 2011–2012 survey wave. 3. Differences in the number and types of diet-related questions asked across survey waves could make it difficult for comparisons to be made over time. 4. Relatively high cost to access data. 5. Lengthy application process and possible lag time for approval.
	**British Regional Heart Study (BRHS)**	**British Women’s Heart and Health Study (BWHHS)**
**Survey Background**	The BRHS is a cohort study, established in 1978–1980, which explores the factors associated with heart disease, hypertension and stoke amongst 7735 middle-aged men (40–59 years at recruitment) recruited from General Practices (GPs) in 24 towns in England, Scotland and Wales.	The BWHHS is a cohort study, established in 1999 as a complement to the BRHS. The study follows 4286 women, aged 60 years and over (at recruitment) from 24 General Practices (GPs), in 23 towns in England, Scotland and Wales
**Survey Design and Methodology**	Longitudinal Cohort study. Almost 8000 middle-aged men who were selected at random from one GP in each of the 24 towns, were examined over the 1978–1980 period. Self-completed health and lifestyle questionnaires and clinical assessments/examinations (inclusive of anthropometric measurements) completed at baseline (1978–80). Follow-up self-completed questionnaires were completed in 1985,1992,1996,1998–2000, 2003,2005,2007,2010–12, 2014,2015, 2016,2017 and 2018. A review of GP records (including all hospital and clinic correspondence) was also conducted bi-annually. A clinical re-examination was done in the 1998–2000 wave. Participants were also given a self-completed activity survey questionnaire and asked to wear an activity monitor and keep a 3-day activity diary in 2010, 2011, 2012, 2013, 2015 and 2017.	Longitudinal Cohort study. Almost 8000 middle-aged women were randomly selected from 24 GPs, in 23 towns from 1999 to 2000. Self-completed health and lifestyle questionnaires, and nurse administered interviews and medical examinations were completed at baseline (1999–2000). A review of GP records (including all hospital and clinic correspondence) was completed at baseline and in 2002, 2004, 2007, 2011–12 and 2016–17. Self-completed health and lifestyle questionnaires were completed in 2003, 2007 and 2010–2011. Participants were also given a self-completed activity survey questionnaire and asked to wear an activity monitor/belt and keep a 3-day activity diary in 2010–2011.
**Target population and level of geography covered**	Middle-aged men aged 40–59 years (at recruitment) who resided in 24 towns across England, Scotland and Wales.	Middle-aged women aged 60 years and over (at recruitment) from 23 towns across England, Scotland and Wales.
**Type of dietary assessment used**	Food frequency questionnaire (FFQ)	Food frequency questionnaire (FFQ)
**Primary users of diet-related data**	Academics/Researchers.	Academics/Researchers.
**Data Accessibility/Availability**	Data accessible through University College London	Data accessible through University College London
**Types of variables captured**	Socio-demographic information (age, sex), health status, consumption of fruits and vegetables, fish, meat, bread and a host of other foods which vary across the survey waves.	Socio-demographic information (age, sex), consumption of fruits, vegetables, cheese, milk, red meat and other foods which vary across the survey waves.
**Cost to access**	Unknown (Information inaccessible at time of assessment).	Unknown (Information inaccessible at time of assessment).
**Key features/potential benefits**	1. Cohort study data can be used to track changes over time. 2. Data captured could be used to explore relationships between diet, cardiovascular disease and associated chronic diseases.	1. Cohort study data can be used to track changes over time. 2. Data captured could be used to explore relationships between diet, cardiovascular disease and associated chronic diseases.
**Key considerations**	1. Study not representative English population. Study only captures middle-aged men from 24 towns across sections of Scotland, England and Wales. 2. Differences in the number and types of diet-related questions asked across survey waves could make it difficult for comparisons to be made over time. 3. Based on the current age-group of participants, the study is now primarily focused on issues surrounding population ageing.	1. Study not representative English population. Study only captures middle-aged women from 23 towns across sections of Scotland, England and Wales. 2. Differences in the number and types of diet-related questions asked across survey waves could make it difficult for comparisons to be made over time.

## Results

Overall, 17 surveys (5 repeated cross-sectional and 12 longitudinal) were identified and deemed relevant for inclusion within this paper (Table 1). The five repeated cross-sectional surveys were the Living Cost and Food Survey (LCFS), Active Lives Survey (ALS), National Diet and Nutrition Survey (NDNS), Health Survey for England (HSE) and Food and You, all of which were accessible via the UK Data Service (Table 1). The Centre for Longitudinal Studies (CLS) and Understanding Society were the primary institutions responsible for collecting, managing and disseminating data related to the British Cohort Study 1970 (BCS70), Millennium Cohort Study (MCS) and Understanding Society, respectively. However, these were also the only longitudinal surveys which were accessible through the UK Data Service. The nine remaining longitudinal surveys assessed (the Southampton Women’s Survey (SWS), Born in Bradford (BiB), Avon Longitudinal Survey of Parents and Children (ALSPAC), UK Women’s Cohort Study (UKWCS), European Prospective Investigation into Cancer and Nutrition (EPIC Norfolk/Oxford), UK Biobank, Whitehall II, British Regional Heart Study (BRHS) and British Women’s Heart and Health Study (BWHHS)) were primarily accessible through the respective academic and research institutions listed in Table 1. Table 1 provides a detailed summary of each of the 17 surveys reviewed, inclusive of their key features/potential benefits and some key considerations which researchers should note, if or when using any of the following surveys to conduct secondary data analysis.

The HSE remains the primary source of information used by the English Government to monitor and assess changes in the overall health and lifestyle of children (0–15 years) and adults (16 years and over) living in England. Although a sports and recreation survey, the ALS captured annual fruit and vegetable consumption for over 198,000 persons (aged 14 years and over) living in England. The NDNS, on the other hand, is currently the only annual, nationally representative survey which provides detailed information on all foods and beverages consumed by persons 18 months of age and older. Food and You was the only repeated cross-sectional survey which was not conducted annually, but every 2 y (bi-annually).

Of the 12 longitudinal surveys assessed, five (SWS, BiB, BCS70, ALSPAC and MCS) were birth cohort surveys which followed the same group of individuals from birth through to adulthood (Table 1). With the exception of Understanding Society, the remainder of the longitudinal surveys reviewed (UKWCS, EPIC, UK Biobank, BRHS, BWHHS and Whitehall II) were primarily focused on exploring the relationship between diet and health outcomes such as cancer and heart disease, amongst middle- aged persons (aged 35 years and over at the time of recruitment). Understanding Society was the only large-scale, multi-topic longitudinal study, which followed individuals across all age groups (children and adults), living in over 40,000 households in the UK, on an annual basis. As such, one of its key features was its large annual sample size and its national representativeness.

In terms of dietary assessment methods used, the Food and You, BiB, BRHS, BWHHS, MCS and Whitehall II used Food Frequency Questionnaires (FFQs) solely, whereas the SWS, ALSPAC, UKWCS and EPIC used a combination of methods (inclusive of FFQs, across different survey waves). A key feature of the LCFS was the availability of two-week expenditure diaries which captured purchased quantities of food and drink. However, it should be noted that the survey does not capture foods actually consumed by individuals, but rather household food purchasing and expenditure. Understanding Society primarily captured the frequency of fruit and vegetable consumption using a brief dietary instrument. Besides their large annual sample sizes, the HSE and ALS captured the consumption of fruits and vegetables using a single 24-h shortened dietary instrument/screener. The NDNS’ consistent use of the food diary assessment method across the survey waves was a feature which set it apart from the remainder of the surveys which used FFQ, shortened dietary screener instruments, 24-h diet recalls or a combination of these methods across the different survey waves. The use of this method meant that the survey provided detailed information, including nutrient content and portion size, on all foods and beverages actually consumed by individuals, over a four-day period.

Besides methodological changes to the NDNS, and LCFS noteworthy changes to the type and number of diet-related questions asked across the survey waves were observed for 13 of the 17 surveys reviewed (HSE, Food and You, BIB, Understanding Society, BCS70, ALSPAC, UKWCS, MCS, EPIC, UK Biobank, Whitehall II, BRHS and BWHHS).

Of all the surveys reviewed, nine (SWS, BiB, ALSPAC, UKWCS, Whitehall II, EPIC, UK Biobank, BRHS and BWHHS) were not representative of the general English population, all of which were longitudinal surveys. BiB focused on Bradford in the North of England, whereas SWS and ALSPAC were limited to Southampton and Bristol in South East and South West England, respectively. Besides the study’s focus on middle-aged persons, EPIC Norfolk/Oxford was also limited in terms of its focus on the geographical areas of Norwich, Greater Manchester, Oxfordshire and Buckinghamshire. Data captured in BRHS and BWHHS were not representative of the English population and were limited to middle-aged males and females from only 24 and 23 towns (respectively) across Scotland, Wales and England. Although the UK Biobank followed 500,000 persons across the UK, the survey was focused on middle-aged persons. Overall, BCS70, MCS and Understanding Society were the only longitudinal surveys reviewed which were nationally representative.

## Discussion

The primary aim of this paper was to provide researchers, especially those interested in conducting secondary data analysis, with a detailed overview of 17 major diet-related repeated cross-sectional and longitudinal surveys conducted in England over the past 48 years (1970–2018). Following this review, three broad thematic areas were identified. These included: the overall survey design and the different dietary assessment method(s) used in each survey; methodological changes and general inconsistencies in the type and quantity of diet-related questions posed across and within surveys over time; and differences in the level of geography and target groups captured across the surveys.

### Survey design and dietary assessment methods used

Repeated cross-sectional surveys such as the NDNS, HSE, ALS, LCFS and Food and You, are inherently designed to provide researchers with a snapshot of diet and related behaviours for a particular group of individuals (sample), at a particular point in time. With the exception of Food and You (conducted bi-annually), the remaining repeated cross-sectional surveys were conducted annually. Longitudinal surveys (such as SWS, BiB, Understanding Society, BCS70, ALSPAC, UKWCS, Whitehall II, MCS, EPIC, UK Biobank, BRHS and BWHHS) however, are primarily designed to follow the same group of individuals over an extended period of time or across the lifecourse (in the case of birth cohort studies such the SWS, BiB, BCS70, ALSPAC and MCS). It is possible to pool data from individual survey waves/years for repeated-cross-sectional surveys. This could help to increase the overall sample size (where deemed necessary) and could be a means of exploring possible differences in diet and related behaviours across survey waves. However, because repeated cross-sectional surveys capture a different group of individuals at each survey wave, they may be more appropriate for researchers interested in assessing current diet-related behaviours, than those interested in tracking possible changes amongst the same group of individuals over time.

Aside from survey design, it was known that the dietary assessment method(s) used in all 17 surveys would have inherent strengths and weaknesses, depending on the context in which they are used. Unlike previous studies [18, 19, 22], providing a detailed description of the pros and cons of the different dietary assessment methods used in surveys was not within the scope of this review. Nevertheless, similar to those studies, this review found that the type of dietary assessment method(s) used in surveys is another area researchers should closely consider, especially when trying to decide the secondary data sources(s) most aligned to their research questions. For instance, the LCFS captures data on the amount (quantity) of food and drink purchased by households, via 2 week/14-day expenditure diaries (found in the survey’s Family Food Module). This type of information is particularly useful for persons interested in exploring household-level shopping and eating habits, household-level socio-economic variations in diet [5] or evaluating population level food purchasing-focused interventions [2]. Researchers in the Department for Environment, Food and Rural Affairs (DEFRA) rely on LCFS data to calculate cost of living indices and to produce the Government’s annual Family Food Report, which provides estimates of nutrient content and statistics on household food purchases by food type. Although beneficial in these circumstances, because the LCFS is an expenditure survey, its design and focus are not the diet of individuals. Whilst it is possible to use expenditure data (as captured in the LCFS) to estimate the quantity of food consumed and the nutrient intake of individuals within households (proxy measure), this is mostly done in low resource settings, specifically in countries which have limited diet-related data other than that captured in household expenditure surveys [7]. Given that the LCFS is not the only source of diet-related data in England, researchers interested in exploring the actual consumption of individuals and potential demographic and socio-economic differences (e.g. age, sex, educational attainment) in diet in England (using repeated cross-sectional survey data), should consider more appropriate surveys such as the NDNS or others, which have data on the actual diet of individuals.

The NDNS’ consistent use of the food diary assessment method across the nine survey waves (nine waves were completed at the time of this review/assessment) meant that the survey captured detailed information on all foods and beverages actually consumed by individuals, over a four-day period. A key feature of the food diary method is that recording of data is done at the time of consumption, which helps to reduce recall bias or the reliance on memory and improves the quality and accuracy of data collected [22]. Respondents are trained to estimate and record amounts consumed using household measures (e.g. one tablespoon of baked beans) and photographs included in the survey. This type of data could be useful to researchers interested in fully exploring the overall diet, nutrients or portion sizes (not only single food groups such as fruits and vegetables) of individuals living in England and possible socio-demographic differences. However, the food diary method, although beneficial, requires significant financial, physical and human resources to implement, especially on an annual basis, and requires that survey participants be literate and committed to completing the entire process [7, 22]. As a result, individuals with low levels of literacy and those from lower socio-economic groups may be under-represented.

Another key consideration is that the NDNS currently targets 1000 persons (500 adults and 500 children) annually, across the entire UK (England, Scotland, Northern Ireland and Wales). Although customary for surveys which use the food diary method, the survey’s relatively low annual sample size could be seen as a limitation. Nevertheless, the pooling of data across the survey waves is one means of increasing the overall sample size and a possible workaround for researchers desirous of investigating diet across the survey waves. Similar to the NDNS, a key feature of the BCS70 was the availability of food diary data for the 1986 and 2016 survey wave. The use of this method meant that diet-related information captured was detailed and as a longitudinal survey, interested researchers could possibly assess differences or changes in the diet of cohort members over time. However, researchers keen on accessing BCS70 food diary data should note that data for both the 1986 and 2016 waves were being processed at the time of assessment and the expected date of release is yet to be determined.

The traditional 24-h diet recall method captures all foods and beverages consumed the preceding day, ideally, over multiple or repeated assessment periods. Dietary screeners or shortened instruments, however, only assess one or two nutrients/food groups, such as fruits and vegetables or calcium/dairy products [7, 19]. The UK Biobank was the only survey in which 24-h diet recalls were conducted on four separate occasions over a 10-day and 16-month period, respectively. Conversely, respondents in the HSE and ALS were asked to recall their consumption of fruits and vegetables, over a single 24-h period. This meant that a brief dietary assessment instrument (screener) was used in both surveys, and not the traditional 24-h diet recall method as initially assumed. The traditional 24-h diet recall method is beneficial in that it provides more precise estimates of nutrients/food and estimates which are more representative of usual dietary consumption. Given that this method captures all foods and beverages consumed over repeated assessment periods, it may be useful to researchers interested in exploring total diet, rather than just key food groups such as fruit and vegetables. The fruit and vegetable screener used in the HSE and ALS may be more beneficial to researchers interested in assessing current adherence to the national “5-A-Day” (fruit and vegetable) dietary target or those interested in exploring the association between fruit and vegetable consumption, physical activity and related health outcomes/chronic diseases. However, because the HSE and ALS only captured consumption over a single 24-h period, researchers should also bear in mind that day-to-day variations in consumption cannot be accounted for.

FFQs often require that respondents indicate how much and/or how often (e.g. daily, weekly) they consume a set of listed foods over a specific period (e.g. over a week, the last 12 months). Unlike the food diary and 24-h diet recall method, surveys which used FFQs are beneficial as they are usually less burdensome and are able to assess the usual diet of individuals over a long-term period, with the added benefit of larger sample sizes [23]. However, because some FFQs are comprised of a short, pre-selected list of foods, (sometimes referred to as dietary screeners) many aspects of diet are not measured, which may make them prone to systematic errors and not be entirely reflective of diet consumption at the population level [9]. For instance, in Understanding Society, respondents were primarily asked about the number of days in a week they eat fruits and vegetables and the number of portions consumed on those days. Although this captures some elements of diet, the survey’s emphasis on fruits and vegetables may make it inappropriate for researchers more interested in exploring diet in its entirety.

### Methodological changes and changes to survey questions over time

As expected, more than a half of the surveys reviewed either had changes made to the type and number of questions asked and the level of detailed captured over time or the survey design/methodology used. For instance, the NDNS, established in 1992, initially consisted of four separate cross-sectional surveys which captured data for individuals from specific age groups (e.g. persons aged 19–64 years in 2000–2001), 18 months and older, across the 1992–2001 period. However, with the introduction of the rolling programme (the NDNS RP) in 2008, the survey changed from a series of ad-hoc age-group specific surveys, to an annual repeated cross-sectional survey for all age groups. As a result, data captured prior to 2008 may not be easily compared with NDNS RP data, which could affect researchers interested in assessing food consumption in England, especially by age. Besides methodological changes, as expected there were notable changes to the type and number of diet-related questions posed across the survey waves. However, the most noteworthy were those made to the HSE across the survey waves. Prior to 2009, the HSE had a “Fruit and Vegetable Consumption” module in addition to an “Eating Habits” module, which captured the frequency of consumption for at least 12 food items via a FFQ. Food categories included: cheese, red and white meat, fried food, sweets, fizzy drinks, among others. However, since 2009, the HSE only captures data on fruit and vegetable consumption, as it is currently the primary survey used by Public Health England to monitor the Government’s national “5-A-Day” target [17]. Although the survey is currently focused on fruit and vegetable consumption, it should be noted that the “Fruit and Vegetable Consumption” module was completely omitted from the survey in 2012, for all age groups and was omitted in 2014 for persons 16 years and older. These changes could possibly affect researchers interested in monitoring fruit and vegetable consumption specifically for the 2012 and 2014 survey period, as well as persons interested in merging and analysing data across several survey waves, inclusive of the 2012 and 2014 waves.

The rapid and ever-evolving field of nutrition science could possibly explain some of the changes observed in the surveys reviewed over the paper’s 1970–2018 review period. However, it should also be acknowledged that survey content, questions asked over time and the methodology used is ultimately based on the overall purpose and intended use of the survey, and the priorities, interests and needs of survey administrators/Governmental Departments/primary stakeholders, rather than the research interests of researchers/users of secondary data. For instance, although surveys such as the HSE capture some aspects of diet, researchers should recall that the survey’s main purpose or focus is not on diet, but on capturing the overall health status of the population and associated risk factors. Also, changes to the type of survey questions asked and the level of detail captured over time, is heavily dependent on the financial, physical and human resources available. Whilst funders and data collectors are cognisant of some of the general interests and data needs of secondary data users, they are also faced with the tremendous challenge of balancing the needs of primary stakeholders and reducing survey costs and participant burden [15]. Researchers therefore need to be aware and constantly keep abreast of survey changes (such as those highlighted in this paper) and their potential impact (positive or negative) on research and devise workaround strategies needed to meet their unique research needs, as far as possible.

### Geographical areas and groups targeted across the surveys

Another major consideration which researchers should acknowledge is the different geographical areas/regions and target groups captured across the surveys. All repeated cross-sectional surveys reviewed were nationally representative and the BCS70, MCS and Understanding Society were the only nationally representative longitudinal studies reviewed. SWS, BiB and ALSPAC could be beneficial for researchers interested in tracking changes in the diet-related behaviours of cohort members from birth through to adulthood. However, it should be noted that these surveys were only focused on certain regions of England, (specifically Southampton, Bradford and Bristol/Avon Health Authority and surrounding areas, respectively), of interest to the respective survey administrators/academic institutions. Similarly, the UKWCS, Whitehall II, EPIC, BRHS, BWHHS and UK Biobank were not representative of the English population, as they targeted certain groups within the population, such as women, middle-aged persons, middle-class persons, vegetarians or members of the Civil Service. Groups which although of possible interest to some researchers, were specifically aligned to the interests and needs of the administrators/academic institutions responsible for these surveys.

In terms of the repeated cross-sectional surveys reviewed, a key feature of the ALS was its annual sample size of over 198,000 individuals and data at the local authority level. The ALS was the only repeated cross-sectional survey reviewed in which data below the Government Office Region (GOR) level was readily available in survey datasets. The ALS could be especially beneficial to researchers (e.g. public health geographers) interested in exploring diet (fruit and vegetable consumption) and possible variations at the national, regional and sub-regional/local authority level. However, persons interested in accessing data below the regional (GOR) level should note that this information is not included in the general End User License for the HSE, NDNS, LCFS or Food and You survey datasets. This type of information needs to be specially requested and approved, and in some instances (in case of the HSE), at an additional cost, to cover data processing and administration fees. Based on the General Data Protection Regulation (GDPR) and other disclosure guidelines, the UK Data Service has instituted strict measures regarding access to sensitive data (e.g. lower-level/sub-regional geographical data), which could be used to reveal the identity of participants [17]. These are other considerations researchers need to acknowledge when trying to decide the survey(s) best aligned to their unique research questions/interests.

### Strengths and weaknesses of this review

The research presented involved a detailed process to provide researchers, especially those interested in conducting secondary data analysis, with an overview (inclusive of key features and practical considerations) of 17 major diet-related repeated cross-sectional and longitudinal surveys conducted in England over the past 48 years (1970–2018). A major strength is that the findings presented in this paper should save researchers interested in diet-related research, time and well-needed resources in compiling this type of information from scratch. This structure is one that may be easily replicated as a follow-up as resources change, providing a clear template for the evaluation of available sources for secondary data analysis of population diet in England. This review did not discuss new and emerging technology-based dietary assessment methods (e.g. web-based and mobile device applications or the use of “big data”), which is a limitation. However, such methods are still not clearly defined and not comprehensively captured in repositories or widely available for re-use [24, 25]. Also, the surveys reviewed may not be exhaustive of all diet-related surveys conducted in England over the 1970–2018 period. The paper’s focus on longitudinal and repeated cross-sectional surveys meant that surveys conducted only once were not included within this review. Therefore, cross-sectional surveys such as the Low-income Diet and Nutrition Survey (LIDNS) and What about Youth (WAY), conducted in 2003–2005 and 2014–2015 (respectively) were not assessed. The detailed description of the pros and cons of the different dietary assessment methods used in surveys was not within the scope of this review. As a result, the review’s failure to discuss the availability of biomarker data in surveys such as the NDNS and the usefulness of this kind of information for validating self-reported dietary data, was another limitation.

The review process used in this paper was time consuming but was a task which assisted the paper’s Review Team (MC, DS, JB, GM and CV) in identifying the surveys most appropriate for their individual research projects. During this process, the need for a review of the current status of diet-related surveys conducted in England over time was identified, particularly if benefits and the practical considerations to using surveys datasets could be incorporated as part of a review. Although the survey documentation required to conduct the review was readily available online from the UK Data Service, CLOSER, CDRC and the MRC Cohort Directory, to the best of our knowledge, no resource exists which provides a comprehensive list and background on the major repeated cross-sectional and longitudinal surveys in England. Although Rippin et al. [20] previously assessed the current status of nationally representative surveys in Europe, it focused on the 53 countries in the WHO European region and not England specifically. Griffith, O’Connell & Smith [11] noted some benefits and possible limitations of diet-related surveys in England. However, unlike this review, their assessment was limited to only three data sources: the NDNS, LCFS and Kantar Worldpanel. Coleman [6] comprehensively summarised 16 longitudinal surveys conducted in England, over the 2005–2015 period. However, Coleman’s report was not focused on diet-related behaviours because it was intended to provide the Department of Education with the information necessary to plan interventions and meet the educational needs of children and young persons under age 19. This review has helped to fill this gap in the literature. Overall, the findings presented indicate that although several diet-related surveys have been conducted over the years, each with their own unique benefits/features, there are still several practical considerations which researchers should note when considering the survey(s) best suited to their research interests.

## Conclusion

Diet-related surveys continue to be the major source of information used by researchers and policymakers to assess dietary patterns, monitor trends over time, evaluate the success/failure of interventions and identify potential inequalities. It is highly unlikely that any survey conducted will meet all the needs of researchers. Additionally, data-related challenges faced by researchers will inevitably vary based on the nature of the research question(s). Regardless, it is still vital that researchers clearly define their research question(s), critically analyse the secondary survey data available (as done in this paper), gain a full understanding of the unique survey characteristics and note key considerations, before delving into data sets. In some instances this may mean that initial research questions may have to be modified or refined, where data of interest may be limited, unavailable, inconsistently captured across survey waves, captured/defined in a manner not befitting to research questions or perhaps too costly to access based on financial constraints. Although not ideal, this is one possible strategy which may help to save time and money and could help researchers to make the best use of the data currently available.

Enhanced communication and engagement between data collectors, data users (existing and new/emerging), data repositories, funding agencies and policy makers could help to ensure that the data being collected is appropriate and cost-effective to inform policy and intervention development. However, researchers using secondary data must acknowledge that change is inevitable and that the type of dietary assessment used, the type of questions included and the level of detail captured in surveys over time, ultimately depends on the priorities and interests of primary stakeholders, the overall purpose and intended use of the survey, and the financial, physical and human resources available. With the increasing prevalence of sub-optimal diet and as research budgets continue to tighten, funding agencies, governments and research institutions are constantly having to consider new, cost-effective and creative methods (e.g. big data and digital technology) of maintaining existing repeated cross-sectional and cohort studies, retaining survey participants and overcoming geographical constraints. In light of these challenges, researchers therefore need be cognisant of these practical considerations, and as far as possible, make every effort to make “effective, proper and good use” of the secondary data currently available, in order to conduct the research necessary for the creation of more evidence-based diet-related policies and interventions in England.

## Data Availability

Not applicable.

## Abbreviations

ALS: Active Lives Survey
ALSPAC: Avon Longitudinal Study of Parents and Children
BCS70: British Cohort Study 1970
BiB: Born in Bradford
BRHS: British Regional Heart Study
BWHHS: British Women’s Heart and Health Study
CDRC: Cohort Directory and the Consumer Data Research Centre
CLOSER: Cohort and Longitudinal Studies Enhancement Resources
CLS: Centre for Longitudinal Studies
CV: Christina Vogel
DEFRA: Department for Environment, Food and Rural Affairs
DS: Dianna Smith
EFS: Expenditure and Food Survey
EPIC: European Prospective Investigation into Cancer and Nutrition
FFQ: Food frequency questionnaire
FSA: Food Standards Agency
GDPR: General Data Protection Regulation
GM: Graham Moon
GOR: Government Office Region
GP: General Practitioner
HSE: Health Survey for England
JB: Janis Baird
LCFS: Living Cost and Food Survey
LIDNS: Low-income Diet and Nutrition Survey
MC: Monique Campbell
MCS: Millennium Cohort Study
MRC: Medical Research Council
NDNS: National Diet and Nutrition Survey
NHS: National Health Service
PHE: Public Health England
RP: Rolling Programme
SWS: Southampton Women’s Survey
UCL: University College London
UK: United Kingdom
UKWCS: UK Women’s Cohort Study
WAY: What about Youth
WHO: World Health Organisation
